# Harnessing electromagnetic data for tsunami source estimation: a comprehensive review

**DOI:** 10.1098/rsta.2024.0082

**Published:** 2024-12-02

**Authors:** Toshitaka Baba, Zhiheng Lin, Takuto Minami, Hiroaki Toh

**Affiliations:** ^1^ Graduate School of Technology, Industrial and Social Sciences, Tokushima University, Tokushima, Japan; ^2^ Center for Data Assimilation Research and Applications, Joint Support-Center for Data Science Research, Tachikawa, Japan; ^3^ Department of Planetology, Faculty of Science, Kobe University, Hyogo, Japan; ^4^ Data Analysis Center for Geomagnetism and Space Magnetism, Graduate School of Science, Kyoto University, Kyoto, Japan

**Keywords:** electromagnetic observation, tsunami observation, motional induction, tsunami inversion

## Abstract

Ocean-bottom pressure gauges are widely used for tsunami observations due to their established accuracy and stability. Recent advancements reveal that the magnetic field fluctuates when a large tsunami passes over the ocean, suggesting potential alternatives to pressure gauges in the form of ocean-bottom electromagnetometers (OBEMs). This article offers a comprehensive synthesis of recent findings concerning tsunami magnetic fields and their utility in tsunami source estimation. In addition, we scrutinize the effectiveness of tsunami observations employing OBEMs. Despite the promise of electromagnetometers, it is worth noting that the background noise inherent in electromagnetic observations tends to be approximately 10 times greater than that of pressure observations within the critical tsunami frequency bands. The Earth’s magnetic field sporadically disrupts tsunami magnetic fields, presenting a potential limitation to the utility of electromagnetometers in tsunami detection when compared with pressure gauges. Nevertheless, our investigation underscores the potential of electromagnetic observations in detecting tsunamis propagating over the ocean at magnitudes of a few centimetres. An invaluable advantage of electromagnetometers over pressure monitoring lies in their capability to observe tsunami velocity fields, suggesting a promising avenue for further research and development in tsunami observation technology.

This article is part of the theme issue ‘Magnetometric remote sensing of Earth and planetary oceans’.

## 1. Introduction

Tsunamis are generated by seafloor deformation caused by earthquakes and submarine/coastal landslides. Gravitational force drives tsunamis over the ocean at a speed of $\sqrt{gH}$ where g is the gravitational acceleration and H is the water depth. Complex bathymetry causes the refraction and diffraction of tsunamis. Reflected tsunamis strike the coast again following refraction and diffraction effects. Because tsunamis are long waves with weak energy decay, they can cross oceans and cause damage to distant coasts. Tsunamis are more destructive than short waves of the same height, and tsunamis with a few metres of flow depth can sweep wooden houses away. Tsunamis of a flow depth of 0.5 and 0.2 m can wash away cars and people, respectively. Tsunamis pose significant global challenges, exemplified by devastating events, such as the 2004 Sumatra earthquake (e.g. [1–3]), 2010 Chile earthquake (e.g. [4–6]) and 2011 Tohoku earthquake (e.g. [7–9]).

**Figure 1.**
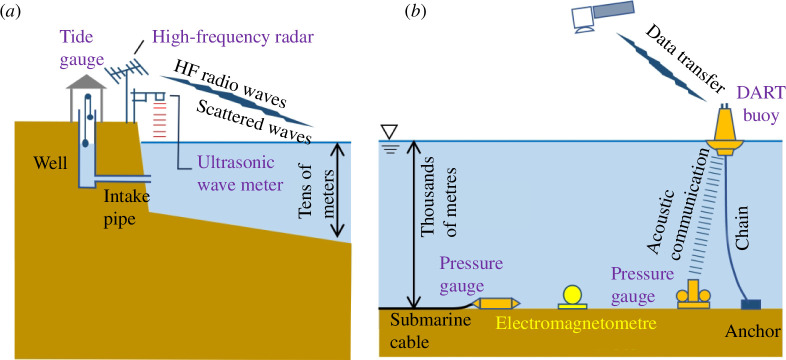
Schematic diagram showing (*a*) coastal and (*b*) deep-ocean tsunami observation systems.

Tsunami water height observations have been conducted using tide gauges on the coast (figure 1*a*). Tide gauges have been used to observe the rise and fall of a float on the water surface in a tidal well connected to the sea via an intake pipe. The intake pipes reduce the short-period fluctuations of the sea surface caused by wind waves. The response of intake pipes varies depending on their diameter and length. This observation method is simple, robust and reliable; thus, stable data can be acquired over a long period of time. However, tidal wells have upper and lower limits for observing tsunami heights, and the pipe response characteristics are subject to change over a longer time period because of the accumulation of seaweed and sand in the pipes; therefore, tide gauges cannot detect tsunamis within wave periods shorter than several minutes.

In recent years, tsunami observations using ocean-bottom pressure (OBP) gauges based on the relationship between seafloor water pressure and ocean depth have become common (figure 1*b*). OBP tsunami observations offer high accuracy and precision, capable of detecting tsunamis in the open ocean with amplitudes as small as one millimetre. They present a versatile deployment option across the entirety of the ocean, contingent upon budgetary considerations. The frequency range for the OBP observations is wide. The National Oceanic and Atmospheric Administration (NOAA), USA operates the Deep-Ocean Assessment and Reporting of Tsunamis (DART) system [10,11]. A DART station consists of an OBP gauge and a sea-surface buoy. OBP data are transmitted in real time to the shore via underwater acoustic and satellite communications. DART stations have been deployed in oceans around the Pacific Rim, Hawaii, India and in the Atlantic Ocean. OBP gauges connected by submarine cables are standard in Japan. Electricity can be provided to ocean-bottom systems through cables, and data can be acquired at a high sampling rate in real time. Two large-scale observatories [12,13] equipped with ocean-bottom seismometers and OBP gauges are now operational.

Other methods for observing tsunamis include ultrasonic wave meters (e.g. [14]), global positioning system tsunami meters [15,16], oceanic high-frequency radars [17,18] and satellite altimetry ([3,19,20]; figure 1). Attempts have also been made to detect tsunamis using shipboard automatic identification systems [21–23]. However, tsunami observations are sparse and insufficient. Further improvements in the observation networks and new methods are required to mitigate tsunami disasters.

**Figure 2.**
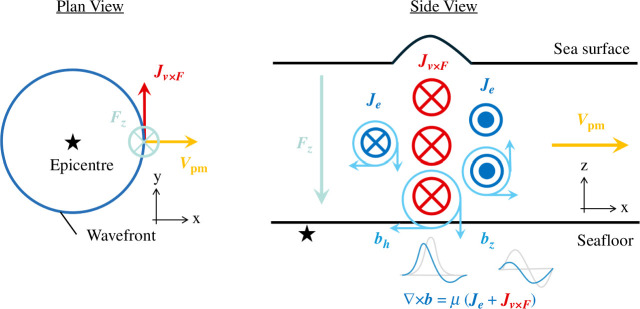
Electromagnetic field in the ocean associated with tsunami propagation. As the tsunami wavefront moves radially away from the epicentre, it generates a circular curtain of electric current (***J*_v × F_**) within the ocean by the coupling of tsunami particle motion (*v*_pm_) with the downward component of the geomagnetic main field (*f*_z_). The electromagnetic induction causes the induced electric current (*j*_e_).The total electric current (***J*_e _*× J*_v×F_**) generates the secondary geomagnetic field (*b*) as the tsunami magnetic field. For further details, refer to Minami *et al*. [26].

Electromagnetometers have generally been used to probe the distribution of underground electrical conductivity. Ocean-bottom electromagnetometers (OBEMs), however, have recently been found to be capable of observing tsunamis [24]. Seawater, being a highly conductive medium, facilitates the flow of electric currents when it is moved by tsunamis within the background magnetic field, known as the geomagnetic main field. This phenomenon aligns with the principles of ‘motional induction’ as originally conceptualized by Faraday [25], representing the manifestation of the ocean acting as a dynamo. The induced electric current in the seawater generates a secondary magnetic field (figure 2). Toh *et al*. [24] termed the secondary magnetic field as the ‘tsunami magnetic field’. Since then, there have been several reports on tsunami magnetic field observations to demonstrate the advantages and disadvantages of tsunami observations using OBEMs (e.g. [27–29]). Minami [30] provided a comprehensive review of tsunami magnetic studies up to the mid-2010s. However, significant advances have been made in applying the tsunami magnetic field to tsunami source estimation since Minami [30]. Therefore, this study aimed to review observations and tsunami source estimations using new observatories. Furthermore, we conducted a comparative study of OBEM- and OBP-derived tsunami waveforms observed at the same location to discuss the capabilities of high-precision tsunami observations.

## 2. Theory of the tsunami magnetic field

The governing equations for the magnetic field induced by the motion of seawater in the Earth’s magnetic field are given in the frequency domain as follows:


(2.1) $$\left(\nabla^{2}+i\omega \sigma \mu_{0}\right)b=-\sigma \mu_{0}\nabla \times \left(v\times F\right),$$

where $b=(b_{x},b_{y},b_{z})$, $v=(v_{x},v_{y},v_{z})$ and $F=(F_{x},F_{y},F_{z})$ are the generated tsunami magnetic field, seawater flow velocity during the tsunami, and external magnetic field, respectively. The local Cartesian coordinates were oriented north (x), east (y) and vertically downward (z). ω, σ and $\mu_{0}$ are the angular frequency of the temporal fluctuation in concern, electrical conductivity, and magnetic permeability in vacuum, respectively. In equation (2.1), b and v are assumed ω-harmonic $\left(\propto e^{-i\omega t}\right)$, and F is assumed to be spatio-temporally constant for the analysis of a single site. Tyler [31] obtained an analytical solution for the water level fluctuation from a tsunami magnetic field assuming linear long-wave equations. Minami *et al*. [32] obtained the following relationships in the horizontal flow velocity using linear dispersive equations with assumptions of a flat seabed and a constant tsunami propagation direction:


(2.2) $$\left\{ \begin{array}{l} v_{y}=\eta \omega \frac{cosh\;\left[k\left(z-H\right)\right]}{sinh\;\left(kH\right)}e^{i\left(ky-\omega t\right)},\\ v_{z}=-i\eta \omega \frac{\sinh\;\left[k\left(z-H\right)\right]}{\sinh\;\left(kH\right)}e^{i\left(ky-\omega t\right)}, \end{array}\right.$$

where η, k and H are tsunami wave height, wavenumber and water depth, respectively. Note that *z* is downward positive and that the positive y direction is in the direction of tsunami propagation. Assuming that the Earth is electrically homogeneous beneath the flat seafloor, we obtained analytical solutions for equations (2.1) and (2.2) within the ocean layer:


(2.3) $$\eta \left(\omega \right)=\frac{b_{z}\left(\omega ,z\right)}{C\left(\omega ,z\right)},\;and$$

(2.4) $$C\left(\omega ,z\right)=C_{1}e^{a_{s}z}+C_{2}e^{-a_{s}z}+\frac{\omega kP}{\sinh\;\left(kH\right)}\cdot \left[iF_{Z}\;\cosh\;\left[k\left(z-H\right)\right]-F_{y}\;\sinh\;\left[k\left(z-H\right)\right]\right],$$

where $a_{s}=\sqrt{k^{2}-i\omega \mu_{0}\sigma_{s}}$, $a_{f}=\sqrt{k^{2}-i\omega \mu_{0}\sigma_{f}}$ and $P=\sigma_{s}\mu_{0}/(k^{2}-a_{s}^{2})$, and where $\sigma_{s}$ and $\sigma_{f}$ are the conductivities of seawater and the sub-seafloor semi-infinite homogeneous medium, respectively. $C_{1}$ and $C_{2}$ are given by


$$C_{1}=\frac{\left(k+a_{s}\right)\cdot \left[Pk\frac{\omega }{\sinh\;\left(kH\right)}\cdot \left(kF_{y}-a_{f}F_{z}\right)\right]-\left(a_{f}-a_{s}\right)e^{-a_{s}h}\cdot \left[-Pk^{2}\cdot \left(F_{y}+iF_{z}\right)\cdot \left(\frac{\omega }{\tanh\;\left(kH\right)}+\omega \right)\right]}{\left(k+a_{s}\right)\left(a_{f}+a_{s}\right)e^{a_{s}h}-\left(k-a_{s}\right)\left(a_{f}-a_{s}\right)e^{-a_{s}h}},$$

$$C_{2}=\frac{\left(k-a_{s}\right)\cdot \left[Pk\frac{\omega }{\sinh\;\left(kH\right)}\cdot \left(kF_{y}-a_{f}F_{z}\right)\right]-\left(a_{f}+a_{s}\right)e^{a_{s}h}\cdot \left[-Pk^{2}\cdot \left(F_{y}+iF_{z}\right)\cdot \left(\frac{\omega }{\tanh\;\left(kH\right)}+\omega \right)\right]}{\left(k-a_{s}\right)\left(a_{f}-a_{s}\right)e^{-a_{s}h}-\left(k+a_{s}\right)\left(a_{f}+a_{s}\right)e^{a_{s}h}}.$$

After applying a Discrete Fourier Transform (DFT) to the magnetic time series data, equations (2.3) and (2.4) can be used to convert the vertical component of the tsunami-generated magnetic field to sea-surface fluctuation (e.g. [33]) at each frequency by using a set of (k,ω) that meets the tsunami dispersion relationship of $\omega^{2}=gk\tanh\;\left(kH\right)$. The inverse DFT of the obtained frequency-domain wave height provided a time series of sea surface fluctuations at an OBEM site, even in the absence of an OBP.

## 3. Tsunami waveform inversion method

Satake [34] developed an inversion method to estimate the fault movement from tsunami waveform data. The linearity of tsunamis allows for the superposition of tsunami waveforms calculated from discretized unit faults. We determined a pair of optimal coefficients that multiplied the tsunami waveforms and superimposed them to reproduce the observed waveforms. The estimated coefficients indicate the slip distribution on the fault plane. The linear two-dimensional tsunami equations are as follows:


(3.1) $$\begin{array}{c} \frac{\partial v_{x}}{\partial t}=-g\frac{\partial \eta }{\partial x}, \end{array}$$

(3.2) $$\begin{array}{c} \frac{\partial v_{y}}{\partial t}=-g\frac{\partial \eta }{\partial y}, \end{array}$$

(3.3) $$\begin{array}{c} \frac{\partial \eta }{\partial t}=-\left(\frac{\partial Hv_{x}}{\partial x}+\frac{\partial Hv_{y}}{\partial y}\right). \end{array}$$

These equations assume that tsunamis are long waves in which water particles in a column from the sea surface to the sea bottom move at the same horizontal velocity ($v_{x},v_{y}$). η is the sea surface displacement caused by the tsunami, H is the water depth under calm conditions and t is the time. Because H is constant during the computation, the tsunami is described by two variables: horizontal velocity and sea surface displacement. Linear dispersive equations that consider the dispersive nature of tsunamis can also be applied to linear tsunami inversions. For the linear dispersive tsunami equations, we added dispersion terms to equations (3.1) and (3.2) to obtain


(3.4) $$\begin{array}{c} \frac{\partial v_{x}}{\partial t}=-g\frac{\partial \eta }{\partial x}+\frac{H}{3}\frac{\partial }{\partial t}\frac{\partial }{\partial x}\left(\frac{\partial Hv_{x}}{\partial x}+\frac{\partial Hv_{y}}{\partial y}\right), \end{array}$$

(3.5) $$\begin{array}{c} \frac{\partial v_{y}}{\partial t}=-g\frac{\partial \eta }{\partial y}+\frac{H}{3}\frac{\partial }{\partial t}\frac{\partial }{\partial y}\left(\frac{\partial Hv_{x}}{\partial x}+\frac{\partial Hv_{y}}{\partial y}\right). \end{array}$$

This is a type of Boussinesq approximation that mimics the general dispersion relation of $\omega^{2}=gk\tanh\left(kH\right)$. The Boussinesq approximation is available for the range of approximately kH<1. The horizontal velocity obtained from the tsunami calculations can be substituted into equation (2.1) to calculate the tsunami magnetic field.

The tsunami inversion procedure began by constructing a fault plane, which was then divided into multiple small subfaults. Seafloor crustal deformations were obtained by applying a unit slip to each subfault (e.g. [35]) and converted to the sea-level change [36]. Sea-level distribution is the initial condition in numerical tsunami simulations using equations (3.1)–(3.3) or equations (3.3)–(3.5) to obtain the tsunami waveforms at the observation points (Green’s functions). In addition to the tsunami equations, the crustal deformation calculation and transformation to the initial sea-level distribution are expressed in linear equations; therefore, a linear inversion is available. Using the observed tsunami waveform (Y) and the Green’s function matrix (G), the observation equation to be solved is as follows:


(3.6) $$\left(\begin{array}{c} Y\\ 0 \end{array}\right)=\left(\begin{array}{c} G\\ \alpha S \end{array}\right)X,$$

where X is the solution to be obtained, which corresponds to the amount of slip for the subfaults. Least-squares methods were used to estimate X**,** which minimized the residual difference between the observed and calculated tsunami waveforms. Non-negative least-squares algorithms [37] are standard for tsunami inversions because the amount of slip should not be negative. S is an *a priori* constraint that stabilizes the solution, such as for spatial smoothness or L2 norm regularization. α is the weight between the observed data and the constraint, which is determined by either Akaike’s Bayesian Information Criterion [38] or cross-validation methods.

## 4. Tsunami magnetic field observations

### (a) SFEMS observations

**Figure 3.**
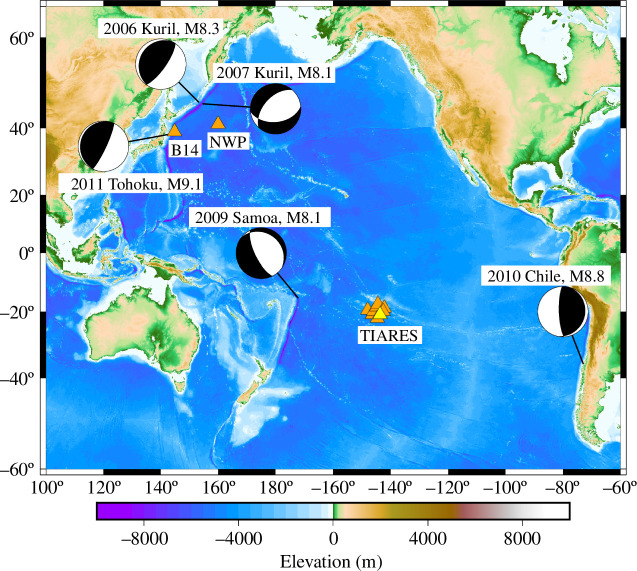
Earthquake events for which tsunami magnetic fields were observed by OBEMs (triangles). The SOC8 station of the TIARES network, shown as the yellow triangle, was equipped with both OBEM and OBP. Earthquake data were downloaded from the Web page of the United States Geological Survey at https://earthquake.usgs.gov/earthquakes/browse/significant.php.

The SeaFloor ElectroMagnetic Station (SFEMS) has been deployed on the deep seafloor of the northwest Pacific Ocean since 2001. An OBEM was in operation at a point in the Northwest Pacific Basin (NWP) from 14 July 2005 to 29 April 2007 (figure 3). The data sampling rate at the NWP station was every 2 min. The data resolutions were 0.01 nT and 64 nV/m for the magnetic and electric fields, respectively. The NWP station achieved the world’s first observations of magnetic fields generated by tsunamis during the 2006 and 2007 Kuril earthquakes [24]. The 2006 Kuril earthquake occurred at the plate interface of the Kuril Trench approximately 800 km northwest of the NWP station [39–42]. The 2007 Kuril earthquake occurred in the subducting plate on the seaward side of the trench axis, slightly southeast of the 2006 epicentre [43]. This earthquake may have been induced by the 2006 Kuril earthquake [39]. The local magnetic field fluctuated synchronously with the tsunamis at the NWP station. The neighbouring magnetometers on land did not observe any similar magnetic field fluctuations at the NWP station. It was clear that the tsunamis caused magnetic field fluctuations, which were confirmed by both theoretical and numerical investigations.

Toh *et al*. [24] also reported the advantages of the OBEM in tsunami observations. A single OBEM observation can be used to estimate the direction of tsunami propagation and the particle motions of seawater caused by tsunamis because the OBEM observes electromagnetic fields in the x, y and z directions. Theoretically, magnetic field fluctuations precede tsunamis [24,26,31,32], which may be helpful in improving existing tsunami early warning systems (e.g. [44–46]).

Kawashima & Toh [43] modelled the 2007 Kuril tsunami at the NWP station by using linear dispersive equations. Based on the calculated tsunamis, they performed three-dimensional magnetic field simulations and concluded that the 2007 Kuril earthquake favoured the rupture of a southeast-trending fault. This was the first study to indicate the capabilities of OBEM data for source estimations.

Shibahara *et al*. [47] performed a joint inversion analysis using tsunami magnetic fields at NWP and OBP data from DART stations and stations around Japan for earthquake slip distributions with respect to the 2006 and 2007 Kuril earthquakes. Green’s functions of the tsunami magnetic field were obtained by solving equation (2.1) using the finite element method and equations (3.3)–(3.5) using the finite difference method. Because the joint inversion used different data types, the observation equation was changed from equations (3.6) and (4.1) as follows:


(4.1) $$\left(\begin{array}{c} Y_{p}\\ Y_{EM}\\ 0 \end{array}\right)=\left(\begin{array}{c} G_{p}\\ \beta G_{EM}\\ \alpha S \end{array}\right)X,$$

where β is the weight between the magnetic field and seafloor pressure data determined by relative data errors. The subscripts *p* and *EM* denote the pressure and electromagnetic data, respectively. The estimated fault models accurately reproduced both the observed pressure and the magnetic field in the Pacific Ocean. Regarding the 2007 event, Shibahara *et al.* [47] concluded that the southeast-trending fault best represented the source of the tsunami based on the agreement of the subsequent waves in the horizontal components of the magnetic field at the NWP. Accordingly, the magnetic data at the NWP proved crucial for source inversion of the 2007 Kuril earthquake.

### (b) TIARES observations

The Tomographic Investigation by the Seafloor ARray Experiment for the Society hotspot (TIARES [27]) was an array-type observation network consisting of nine OBEMs (SOC1−9) installed between 15–25° S and 152–140° W (figure 3). The sampling rate of the OBEMs was 1 min, and the data resolution was the same as that of the NWP. The installation period from March 2009 to November 2010 was short, but two tsunami magnetic fields were successfully recorded for the 2009 Samoa (e.g. [48,49]) and 2010 Chile tsunamis (e.g. [4–6]). One TIARES station (SOC8) was equipped with an OBP and an OBEM. Sugioka *et al*. [29] compared the OBP and OBEM data at SOC8 and found excellent consistency. Furthermore, Lin *et al*. [33] converted the tsunami magnetic field to sea-level change using analytical solutions by Minami *et al*. [32] (equations (2.3) and (2.4)) and Tyler [31]. The OBEM-derived sea-level changes agreed with the OBP-derived sea-level changes. They were also able to exploit the significant phase lead in the Z component of the tsunami magnetic field, which may be useful for early tsunami warning. According to Minami *et al*. [26], the lead time increases linearly with the tsunami wave period.

Yokoi *et al*. [50] performed a joint tsunami inversion using the OBEM- and DART’s OBP-derived sea-level data in the Pacific Ocean for the 2009 Samoa earthquake. The inversion analysis was straightforward because all the data were converted to sea-level fluctuations. The estimated slip distribution explained the tsunami waveform data observed by the OBPs and OBEMs. Yokoi *et al*. [50] utilized the Z-component of the tsunami magnetic field, converted to sea-level fluctuations, to analyse the 2009 Samoa earthquake. By contrast, Shibahara *et al*. [47] directly incorporated all components of the tsunami magnetic fields into their tsunami source inversion for the 2007 Kuril earthquake.

### (c) Observations in the vicinity of the Japan Trench

Ichihara *et al*. [28] have repeatedly collected electromagnetic field data of the landward and seaward slopes of the Japan Trench using the magnetotelluric method for estimating the electrical conductivity structure beneath the seafloor. On 16 November 2010, they deployed OBEMs at two locations, one approximately 30 km west (B13) and the other 50 km east (B14, figure 3) of the Japan Trench. On 11 March 2011, the great Tohoku earthquake occurred near the OBEM stations, generating a devastating tsunami. Ichihara *et al*. [28] successfully recovered the data of the 2011 Tohoku earthquake from the OBEM at site B14, which recorded time variations in three magnetic field components, two horizontal electric field components and two components of the instrumental tilt level at 1 min intervals. The tsunami magnetic field fluctuation in close proximity to the seismic source exhibited an immense scale. Substantial magnetic field fluctuations with peak amplitudes of 12–19, 4–5 and 8–17 nT were observed in the bz, bx and by components, respectively, corresponding to a sea surface rise of 1.4−2.9 m.

The observed magnetic data were continuous without data gaps so that Ichihara *et al*. [28] could mark the onset time of the fluctuation of the total magnetic field. In deep ocean areas, such as the vicinity of the Japan Trench, bz becomes almost in phase (<20 degrees) with the sea-level fluctuation due to the strong self-induction effect [26]. Assuming that the onset time of bz and total magnetic field fluctuation is the arrival time of the tsunami, it allows for estimating the tsunami source location through the backward propagation of the tsunami wavefront from the observation station. The backward propagation technique relies on fundamental physics, where water depth determines tsunami propagation speed (i.e. $\sqrt{gH}$). This method assumes that the time taken for the tsunami to travel from the source to the observation point equals the time it takes to travel from the observation point to the source. If multiple stations record tsunami arrival times, backward-propagated wavefronts converge within an area corresponding to the tsunami source. This is the same principle used to determine the epicentre using seismic waves. This approach, while simpler than other tsunami inversion analyses, effectively constrains the source area due to its reliance on known arrival times and is less susceptible to complexities found in late-arrival waves such as tsunami reflection, refraction and nonlinearities. For instance, Ichihara *et al*. [28] used tsunami arrival time recorded by OBEM and identified the tsunami source region as a narrow band along the Japan Trench, approximately 100 km north of the primary rupture zone, with the source onset estimated to be approximately one minute after the origin of the earthquake. This area coincided with regions of submarine landslides, as suggested by Tappin *et al*. [51]. However, the theory that the bz fluctuation is in phase with the sea-level fluctuation in the deep ocean is valid only when the seafloor can be approximated as flat. Even in this case, the phase lead of tens of degrees is inevitable for bz, while significant phase lead occurs in the horizontal components of tsunami magnetic fields [26]. Therefore, determining the precise tsunami arrival time using OBEM data may necessitate further clarification, especially for steep bathymetry such as the Japan Trench.

## 5. Discussion

**Figure 4.**
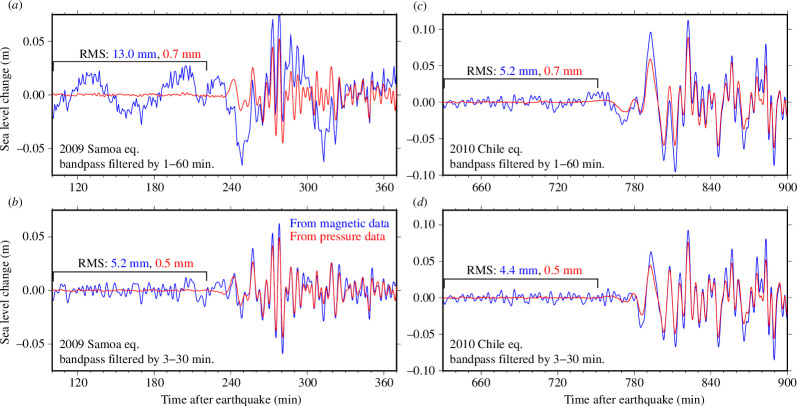
Comparisons of magnetic- (blue) and pressure-derived (red) sea-level fluctuations for the tsunamis caused by the 2009 Samoa (*a,b*) and the 2010 Chile (*c,d*) earthquakes at the SOC8 station of the TIARES network. Background noise levels were estimated using data 2 h (range represented by the horizontal black bar) before the arrival of the tsunami using root mean square (RMS).

The calm nature of the seafloor renders it suitable for observing tsunami-related pressure changes, as water pressure fluctuations due to wind-induced waves with short wavelengths dissipate rapidly with increasing water depth. Our study focuses on assessing the effectiveness of OBEMs for tsunami observations on the seafloor. The OBP observations were more precise than the OBEM observations. The resolution of the seafloor water pressure observation was 0.1 Pa (~0.01 mm), with a sampling rate of 1 s. By contrast, the OBEM resolution (0.01 nT) was approximately equivalent to a digit error of 2.27 mm at the sea level for a water depth of 4000 m and a sampling rate of 1−2 min. Fortunately, one of the TIARES stations (SOC8) had both OBEM and OBP sensors. We compared the OBEM- and OBP-derived tsunami waveforms for the 2009 Samoa and 2010 Chile earthquakes (figure 4). The waveform data were obtained from Yokoi *et al*. [50]. Background noise levels were estimated using data collected 2 h before the tsunami arrival. OBP data revealed background noise levels of 0.5−0.7 mm, enabling the detection of tsunamis with amplitudes of a few millimetres. Conversely, background noise levels were 4.4−13.0 mm for sea-level fluctuations derived from OBEM data, suggesting the capability to detect tsunamis propagating over the ocean with amplitudes of a few centimetres. Although OBEMs can effectively observe tsunamis caused by earthquakes with magnitudes of 8 and above, such as the 2009 Samoa and 2010 Chile earthquakes, even at considerable distances of several thousand kilometres away from the OBEMs, they may not be suitable for detecting micro tsunamis with amplitudes less than a centimetre in the open ocean. The pronounced background noise in the magnetic field-derived sea-level fluctuations was probably related to the low data resolution of 0.01 nT of the OBEMs’ fluxgate magnetometers.

It is worth mentioning that OBP records several types of water pressure fluctuations caused by tsunamis, crustal deformation and seismic waves, including T-phases. The water pressure fluctuations caused by seismic waves are much larger than those caused by tsunamis. However, the propagation velocity sufficiently differs that we can clearly distinguish the pressure fluctuations caused by seismic versus tsunami waves at stations far from the source. Even if the waves overlap at a nearby station, their dominant frequency bands differ, so that appropriate filtering can recover the pressure fluctuations caused by tsunamis. Crustal deformation of earthquakes causes significant static and dynamic water pressure fluctuations. However, the pressure fluctuations are limited to the earthquake source region and do not cause problems at many ocean-bottom stations except at stations above the source.

**Figure 5.**
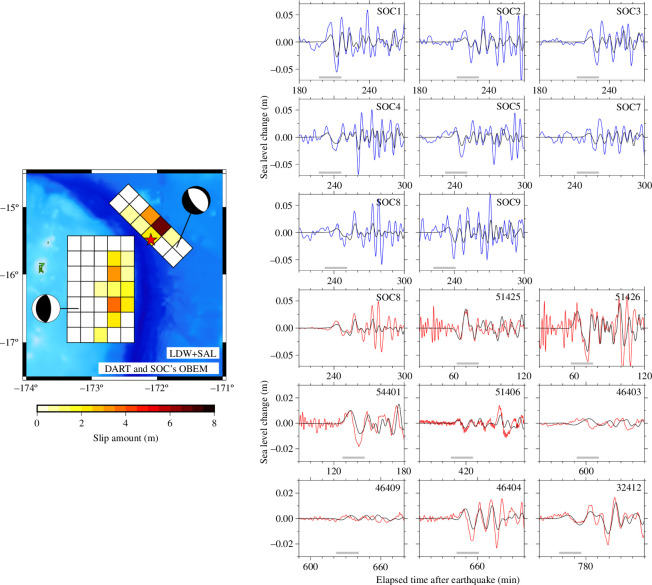
Estimated (left) slip distribution and (right) comparison of observed and synthetic tsunami waveforms at the SOC and DART stations for the 2009 Samoa earthquake [50]. The earthquake was a doublet consisting of two main ruptures on the normal and reverse faults. Observed tsunami waveforms derived from OBEM and OBP are in blue and red, respectively. Waveforms in black were calculated from the slip model. Horizontal grey bars under the waveforms indicate the time windows used in the inversions as containing the first tsunami waves.

The amplitudes of peaks and troughs of tsunami waveforms derived from OBEM data consistently exhibited approximately 20% higher values compared with those estimated from OBP data (figure 4). A similar trend was observed in tsunami inversion results for the 2009 Samoa earthquake (figure 5; [50]). The Samoa earthquake involved nearly simultaneous ruptures of a normal fault in the outer rise and a reverse fault at the plate interface. Yokoi *et al*. [50] successfully derived a reasonable slip distribution on both fault planes through a joint inversion analysis employing OBEM- and OBP-derived tsunami waveforms. While the amplitudes of tsunami waveforms observed at the DART stations closely matched calculated values, those observed at OBEM stations SOC 1−9 markedly exceeded calculated values. Ongoing investigations aim to determine the underlying causes, including potential theoretical deficiencies, inaccuracies in the conversion of tsunami magnetic field data, parameterization problems or instrument characteristics. Addressing this issue is crucial for accurately determining earthquake magnitude and refining source estimations.

The 1 min sampling interval of the OBEMs proves barely adequate for capturing tsunamis with dominant periods ranging from several minutes to tens of minutes. One challenge involves increasing the sampling rate of OBEM observations to precisely record tsunami waveforms, while concurrently reducing battery power consumption for standalone OBEM deployments in deep ocean environments. For example, the DART system incorporates standard and emergency (tsunami) modes for data sampling intervals. The standard and emergency modes collect data every 15 min and 15 s, respectively. The standard mode is switched to the emergency mode when large pressure perturbations are detected.

In the tsunami waveforms bandpass filtered by spanning 1−60 min of the 2009 Samoa earthquake (figure 4*a*), OBEM-derived waveforms exhibited incoherent components for a relatively long period. However, waveforms from the 2010 Chile earthquake (figure 4*c*), showed no such inconsistencies, indicating that the discrepancy in the Samoa waveforms was probably caused by occasional noise induced by perturbations of the Earth’s magnetic field. Thus, it becomes imperative to accurately estimate geomagnetic noise, as its dominant frequency overlaps with the tsunami frequency band.

The tsunami source of the 2009 Samoa earthquake was better constrained by using OBEM data in the inversion analysis of Yokoi *et al*. [50], but this improvement was simply due to the increased number of observations and better station coverage because the authors used only the vertical component of the tsunami magnetic field. The significant advantage of the OBEM over OBP observations lies in its ability to capture the velocity field of tsunamis [29,33]. As mentioned above, Shibahara *et al*. [47] attempted to incorporate the horizontal magnetic component (i.e. the velocity field of the tsunami) of the OBEM into the inversion analysis, highlighting its pivotal role in estimating tsunami sources. They found that the horizontal component of the magnetic field was sensitive to the dispersive waves of the tsunami. This could be a powerful tool for estimating source models, including fault orientations. More research is needed, but we see great potential in utilizing the horizontal component for tsunami source estimations.

The signal of the vertical tsunami magnetic component arrives earlier than the tsunami, which has been predicted by analytical solutions [24,26,31,32] and numerical simulations [26]. The fact that the tsunami magnetic field variation occurs prior to the tsunami is of great interest for improving early tsunami warnings; this, however, was beyond the purpose of this study, which was focused on estimating tsunami sources using OBEM data. Lin *et al*. [33] successfully observed that the vertical component of the tsunami magnetic field fluctuated prior to the sea level change associated with the 2009 Samoa and 2010 Chile tsunamis. In addition, the horizontal component of the tsunami magnetic field arrived even earlier than the vertical component. Although it is necessary to collect more observations of the phase lead, intensive developments of real-time tsunami prediction methods using the phase lead of the tsunami magnetic field appear to be warranted. Real-time tsunami source estimates may not be suitable for early tsunami warnings because of the need for good station coverage surrounding the source area. Predicting coastal tsunamis directly from the phase lead observed by a single OBEM station would be preferable. For this purpose, we can adapt tsunami amplification factors derived from the conventional Green’s law [52,53], regression models [54,55] and data assimilation techniques [56,57] to OBEM data.

## 6. Conclusions

Tsunami magnetic fields have been observed in the Pacific Ocean during seismic events such as the 2006 Kuril, 2007 Kuril, 2009 Samoa, 2010 Chile and 2011 Tohoku earthquakes. The incorporation of OBEM data into tsunami source inversions is a burgeoning field showing steady progress. Analysis of the 2007 Kuril earthquake confirmed the importance of the horizontal component of the tsunami magnetic field in tsunami source estimations [47]. The background noise level, estimated by root mean square, was approximately 0.5 mm for OBPs and 5 mm for OBEMs based on data recorded at SOC8 of the TIARES network. Furthermore, the amplitudes of peaks and troughs in tsunami waveforms converted from the OBEM data were approximately 20% larger than those estimated from OBPs. OBEM-derived tsunami waveforms are occasionally disrupted by geomagnetic noise. Further efforts are needed to improve the observational performance of OBEMs, characterized by higher sampling rates and data resolution, for the detection of moderate and micro tsunamis amplitude smaller than one centimetre in the open ocean.

## Data Availability

This article has no additional data.
